# Pharmacological inhibition of leukotrienes in an animal model of bleomycin-induced acute lung injury

**DOI:** 10.1186/1465-9921-7-137

**Published:** 2006-11-21

**Authors:** Marco Failla, Tiziana Genovese, Emanuela Mazzon, Elisa Gili, Carmelo Muià, Mariangela Sortino, Nunzio Crimi, Achille P Caputi, Salvatore Cuzzocrea, Carlo Vancheri

**Affiliations:** 1 Department of Internal Medicine and Specialistic Medicine, Section of Respiratory Diseases, University of Catania, Catania, Italy; 2 Department of Clinical and Experimental Medicine and Pharmacology, University of Messina, Messina, Italy; 3 Centro per lo Studio e il Trattamento dei Neurolesi Lungodegenti, University of Messina, Messina, Italy; 4 Department of Clinical and Experimental Medicine and Pharmacology, Catania, Italy

## Abstract

Leukotrienes are increased locally in idiopathic pulmonary fibrosis. Furthermore, a role for these arachidonic acid metabolites has been thoroughly characterized in the animal bleomycin model of lung fibrosis by using different gene knock-out settings.

We investigated the efficacy of pharmacological inhibition of leukotrienes activity in the development of bleomycin-induced lung injury by comparing the responses in wild-type mice with mice treated with zileuton, a 5-lipoxygenase inhibitor and MK-571, a cys-leukotrienes receptor antagonist.

Mice were subjected to intra-tracheal administration of bleomycin or saline and were assigned to receive either MK-571 at 1 mg/Kg or zileuton at 50 mg/Kg daily. One week after bleomycin administration, BAL cell counts, lung histology with van Gieson for collagen staining and immunohistochemical analysis for myeloperoxidase, IL-1 and TNF-α were performed.

Following bleomycin administration both MK-571 and zileuton treated mice exhibited a reduced degree of lung damage and inflammation when compared to WT mice as shown by the reduction of:(i) loss of body weight, (ii) mortality rate, (iii) lung infiltration by neutrophils (myeloperoxidase activity, BAL total and differential cell counts), (iv) lung edema, (v) histological evidence of lung injury and collagen deposition, (vi) lung myeloperoxidase, IL-1 and TNF-α staining.

This is the first study showing that the pharmacological inhibition of leukotrienes activity attenuates bleomycin-induced lung injury in mice. Given our results as well as those coming from genetic studies, it might be considered meaningful to trial this drug class in the treatment of pulmonary fibrosis, a disease that still represents a major challenge to medical treatment.

## Background

Idiopathic pulmonary fibrosis (IPF) is the most common interstitial pneumonias of unknown origin and one of the most aggressive interstitial lung diseases. It is characterized by a chronic and progressive course leading to respiratory failure with a median survival under 3 years [1-3]. The pathogenesis of this condition is not entirely understood, but the activation and proliferation of fibroblasts in response to multiple and microscopic episodes of alveolar epithelial injury is believed to be the main event which ultimately leads to extracellular matrix components remodelling, resulting in the irreversible distortion of the lung architecture [4].

A number of studies suggest a causal role for leukotrienes (LT) in lung fibrosis [5]. These are lipid mediators derived by the hydrolysis from membrane phospholipids of arachidonic acid by the phospholipase A2 and 5-lipoxygenase[6]. Leukotriene B_4 _is elevated in the bronchoalveolar lavage of patients with IPF [7,8]. Furthermore cys-LT and LT-B_4 _are increased in lung homogenates of patients with IPF, and the levels of these mediators were found to correlate with the extent of fibrosis in histological sections [9]. Increased LT levels have also been demonstrated in mice lungs following intra-tracheal administration of bleomycin [10].

The leukotrienes pathway has been recently dissected in the bleomycin animal model of lung fibrosis using different genetic backgrounds. Knocking out each of the enzymes involved in the cascade from membrane phospholipids to leukotrienes, such as phospholipase-A_2_, 5-lipoxygenase (LO), as well as LTC_4 _synthase, invariably attenuates fibrosis in mice [11-13]. However, results coming from these genetically altered backgrounds have not been confirmed using a pharmacological approach, so that no data exist actually on the efficacy of selective drugs targeted on the leukotrienes pathway approved today for human use.

This lack of data prompted us to ascertain whether the cysteinyl leukotrienes receptor-1 antagonist MK-571 and the 5-LO specific inhibitor Zileuton were able to affect the inflammatory and fibrosing process that characterize the intratracheal instillation of bleomycin in mice.

## Methods

### Animals

Male CD mice (25–35 g; Harlan Nossan; Italy) were housed in a controlled environment and provided with standard rodent chow and water. Animal care was in compliance with Italian regulations on protection of animals used for experimental and other scientific purpose (D.M. 116192) as well as with the EEC regulations (O.J. of E.C. L 358/1 12/18/1986).

### Experimental groups

Mice were randomly allocated into the following groups:

(i) WT+BLEO group. Mice were subjected to bleomycin-induced lung injury (N = 15), (ii) WT+saline group. Sham-operated group in which saline was administered instead of bleomycin, (N = 15). (iii) MK-571 group. Same as the WT+BLEO group but mice were administered with MK-571 delivered through a subcutaneous implanted Alzet 2002 mini-osmotic pump (Durect Co., Cupertino, CA, USA). This route of administration was preferred over oral administration on the basis of unknown pharmacokinetic properties of MK571 because of constant drug delivery. The pump loaded with 200 μL of a 2.5 μg/μL MK-571 solution in PBS (Cayman Chemical, Ann Arbor, MI, USA) had a release rate of 0.5 μL/hour during the 7 days of the experimental setup, (N = 15). (iv) Sham+MK-571 group. Identical to WT+saline group, except for the administration of MK-571 delivered as described above (N = 15). (v) Zileuton group. Same as the WT+BLEO group but WT mice were administered Zileuton by force-feeding (Sequoia Research Products, Oxford, U.K.) with a 50 mg/kg oral bolus 30 minutes after bleomycin instillation and then daily in the subsequent days (N = 15). The concentration of MK-571 was established on the basis of preliminary experiments starting from what was available on other animal models [14], while zileuton dose and route administration was chosen according to our precedent studies [15]. (vi) Sham+Zileuton. Identical to WT+saline group, except for the administration of zileuton as previously described (N = 15).

### Induction of lung injury by bleomycin

Mice received a single intratracheal instillation of saline (0.9%) or saline containing bleomycin sulphate (1 mg/kg body weight) in a volume of 50 μl and were killed after 7 days by pentobarbitone overdose.

### Measurement of fluid content in lung

The wet lung weight was measured after careful excision of extraneous tissues. The lung was exposed for 48 h at 180°C and the dry weight was measured. Water content was calculated by subtracting dry weight from wet weight.

### Histological examination

Excised lung were taken 7 days after injection of bleomycin, processed as previously described[16], and stained by the van Gieson stain for collagen. The severity of fibrosis was semi-quantitatively assessed according to Ashcroft and co-workers[17]. Briefly, the grade of lung fibrosis was scored on a scale from 0 to 8 by examining randomly chosen fields of the left middle lobe at a magnification of ×100. Criteria for grading lung fibrosis were as follows: grade 0, normal lung; grade 1, minimal fibrous thickening of alveolar or bronchiolar walls; grade 3, moderate thickening of walls without obvious damage to lung architecture; grade 5, increased fibrosis with definite damage to lung structure and formation of fibrous bands or small fibrous masses; grade 7, severe distortion of structure and large fibrous areas; grade 8, total fibrous obliteration of fields. Grades 2, 4 and 6 were used as intermediate pictures between the aforementioned criteria. All sections were scored by a single investigator in a blinded fashion.

### Immunohistochemical localization of IL-1β and TNF-α

IL-1β and TNF-α were determined by immunohistochemistry as previously described [16]. Sections were incubated overnight with anti-IL-1β or anti-TNF-α (Santa Cruz Biotechnology Inc., Santa Cruz, CA, USA) polyclonal antibody (both at 1:500 in PBS, v/v). Specific labelling was detected with a biotin-conjugated goat anti-rabbit IgG and avidin-biotin peroxidase complex (DBA, Milan, Italy). Controls included buffer alone or non-specific, purified rabbit IgG. Immunocytochemistry photographs were assessed by densitometry. By using Optilab Graftek software on a Macintosh personal computer, the assay was performed.

### Myeloperoxidase activity assay

Myeloperoxidase (MPO) activity, an indicator of polymorphonuclear leukocyte (PMN) accumulation, was determined as previously described in lung homogenates. The rate of change in absorbance was measured spectrophotometrically at 650 nm. MPO activity was defined as the quantity of enzyme degrading 1 μMol of peroxide/min at 37°C and was expressed in milli-units per g of wet tissue.

### Bronchoalveolar Lavage (BAL)

Seven days after bleomycin or saline solution instillation, mice were euthanized and the trachea was cannulated. Lungs were lavaged once with 0.5 ml D-PBS (GIBCO, Paisley, U.K.). In >95% of the mice, the recovery volume was over 0.4 ml. Total BAL cells were enumerated by counting on a haemocytometer in the presence of trypan blue. Cytospins were prepared from resuspended BAL cells. A total of 400 cells were counted from randomly chosen high power microscope fields for each sample.

### Materials

Unless otherwise stated, all compounds were obtained from Sigma-Aldrich Company Ltd. (Poole, Dorset, U.K.). All other chemicals were of the highest commercial grade available. All stock solutions were prepared in non-pyrogenic saline (0.9% NaCl; Baxter, Italy, UK).

### Statistical evaluation

All values in the figures and text are expressed as mean ± standard error of the mean (SEM) of N observations. For the in vivo studies N represents the total number of animals studied, dead animals were replaced in further experiments to reach the specified number of observations. In the experiments involving histology or immunohistochemistry, the figures shown are representative of at least three experiments performed on different experimental days. The results were analyzed by one-way ANOVA followed by a Bonferroni post-hoc test for multiple comparisons. A P-value of less than 0.05 was considered significant. Statistical analysis for survival data was calculated by Fisher's exact probability test. For such analyses, p < 0.05 was considered significant.

## Results

**Figure 1:**
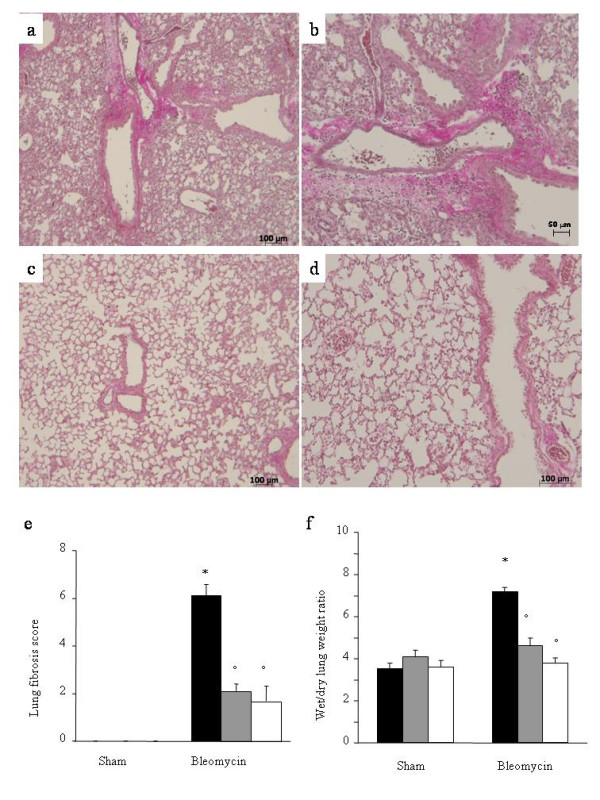
**Effect of leukotrienes pathway pharmacological inhibitionon lung injury**. Van Gieson stain: × 150. The used stain shows collagen in purple. A: Bleomycin alone in WT mice. B: Magnified lung section of Bleomycin alone in WT mice, × 300. C: Bleomycin in MK-571 treated mice. D: Bleomycin in Zileuton treated mice. All showed sections come from the left middle lobe. Each image is representative of at least 3 experiments. E: Lung fibrosis as evaluated by Ashcroft criteria[17]. F: Effect of pharmacological leukotrienes activity inhibition on edema in the lung. Black bar represents control group, grey bar MK-571 group and white bar Zileuton group. Data are means ± SEM from 15 mice for each group. *p < 0.01 versus sham. °p < 0.01 vs. bleomycin.

Histological examination of lung sections revealed significant tissue damage. Thus, when compared to lung sections taken from saline-treated animals, histological examination of WT mice treated with bleomycin were characterized by extensive inflammatory infiltration by neutrophils, lymphocyte and plasma cells extending through the lung epithelium, fibrosis and granulomas were seen in perivascular region (Fig. 1a and 1b). The inhibition of the leukotrienes activity in mice (animals treated with either MK 571 or Zileuton) significantly prevented lung inflammation induced by bleomycin administration (Figs. 1c and 1d, respectively).

Lung fibrosis grading [17] revealed a moderate to severe fibrosis reaction after one week of bleomycin administration, which was significantly reduced in animals treated with MK-571 and Zileuton (6.1+/-0.5 vs. 2.1+/-0.3 and 1.7+/-0.6, p < 0.01, Fig. 1e). Sham treated animals were found to be constantly free from lung inflammation and fibrosis.

**Figure 2:**
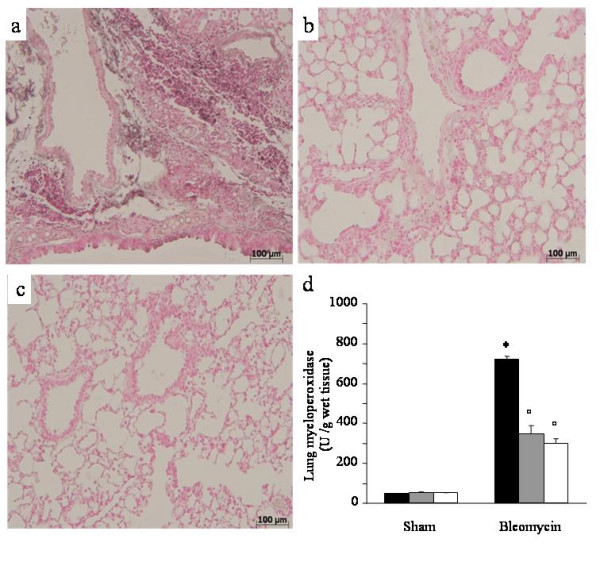
**Effect of pharmacological leukotrienes activity inhibition on lung myeloperoxidase**. Immunohistochemical localization of myeloperoxidase in the lung. A: Bleomycin alone in WT mice. B: Bleomycin in MK-571 treated mice. C: Bleomycin in Zileuton treated mice. Original magnification: 150×. Each image is representative of at least 3 experiments. D: Effect of pharmacological leukotrienes activity inhibition on lung myeloperoxidase activity. Black bar represents control group, grey bar MK-571 group and white bar Zileuton group. Data are means ± SEM from 15 mice for each group. *p < 0.01 versus sham. °p < 0.01 vs. bleomycin.

Bleomycin elicited an inflammatory response characterized by the accumulation of water in lung as an indicator of lung edema, (Fig. 1f) and neutrophils infiltration in the lung tissues in WT-animals. The leukotrienes synthesis inhibition and the receptor blockade in bleomycin treated mice significantly reduced the fluid content (Fig. 1f) and the neutrophil infiltration (Figs. 2d) as evaluated by MPO activity assay. Neutrophil activity was also evaluated immunohistochemically by MPO staining of lung sections, demonstrating a strong alveolar neutrophils infiltration (Fig. 2a). This effect was completely abrogated in MK-571 and Zileuton treated animals (Figs. 2b,c).

**Figure 3:**
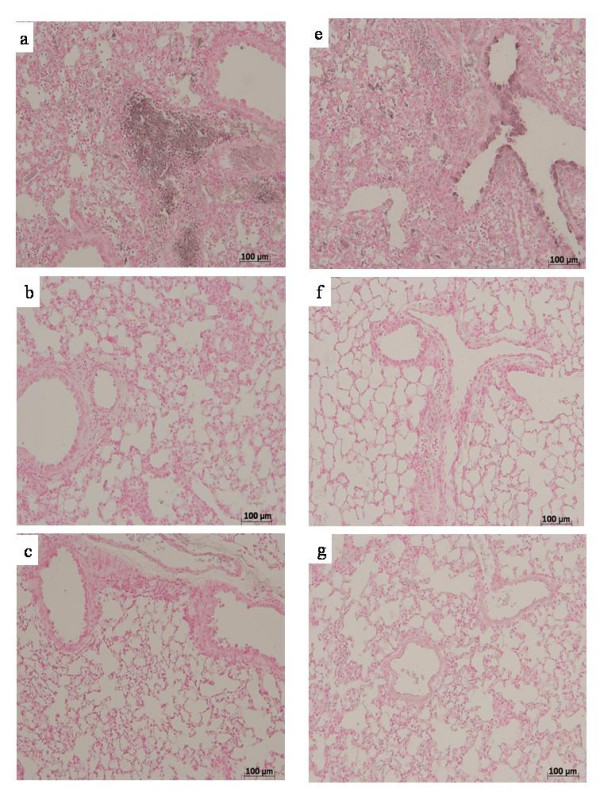
**Effect of pharmacological leukotrienes activity inhibition on lung IL-1 and TNF-α immunostaining**. After bleomycin injection in WT mice, positive staining for IL-1 (A) was localized mainly in inflammatory cells and in vascular endothelium. There was a marked reduction in the IL-1 immunostaining in the lungs of MK-571 group (B) and in the lungs of Zileuton group (C). TNF-α was localized mainly in inflammatory cells and in bronchial epithelium of lungs in the bleomycin group (E). A marked reduction in TNF-α immunostaining in lungs of MK-571 (F) and in Zileuton group (G). Original magnification: 150×. This figure is representative of at least 3 experiments performed on different experimental days.

Immunohistochemical analysis revealed a positive staining for IL-1β mostly in inflammatory cell infiltrate present in the interstitium and in the airspace (i.e. alveolar macrophages) but also in the vascular zone (i.e. vascular endothelium) in bleomycin-group (Fig. 3a). In contrast, no staining for IL-1β was found in the lungs of MK-571 (Fig. 3b) and Zileuton groups (Fig. 3c).

Similarly, a substantial increase in the lung TNF-α staining of bronchial epithelial cells was evident in bleomycin group (Fig. 3e). This effect was reduced in lung sections of MK-571 (Fig. 3f) and Zileuton treated animals (Fig. 3g) caused by bleomycin intratracheal administration. There was no IL-1β or TNF-α staining in lung sections of sham-operated animals.

**Figure 4:**
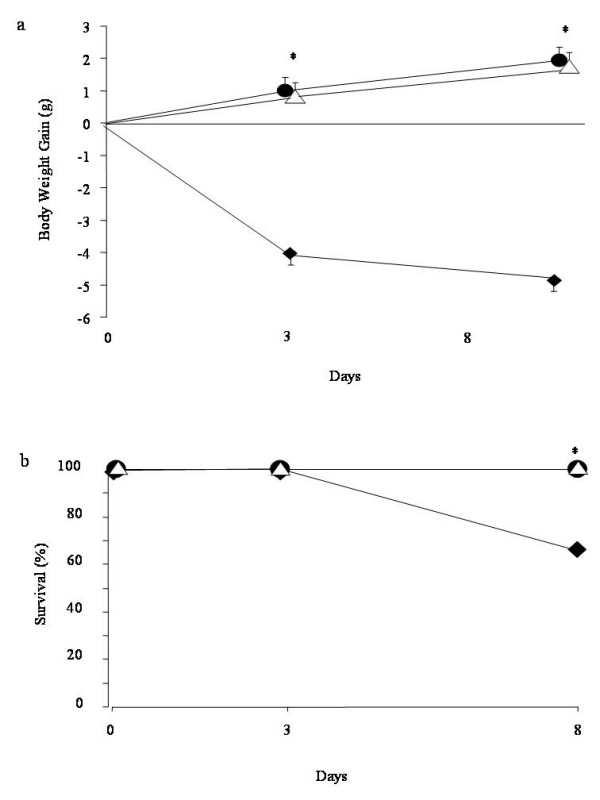
**Effect of pharmacological inhibition of leukotrienes activity on body weight (A) and survival (B)**. ◆ represents bleomycin group, ● MK-571 treated animals and Δ Zileuton treated animals. Data are means ± SEM from 15 mice for each group. *p < 0.01 vs. bleomycin.

The severe lung injury caused by bleomycin administration was associated with a significant loss in body weight and survival (Figs. 4a,b). Leukotrienes synthesis blockade and the receptor antagonism in bleomycin treated mice significantly attenuated the loss in body weight. Bleomycin-treated WT mice developed severe lung injury and 33% of these animals died within one week after bleomycin administration. None of the MK-571 or Zileuton treated animals died after bleomycin instillation within the one week period of study.

**Figure 5:**
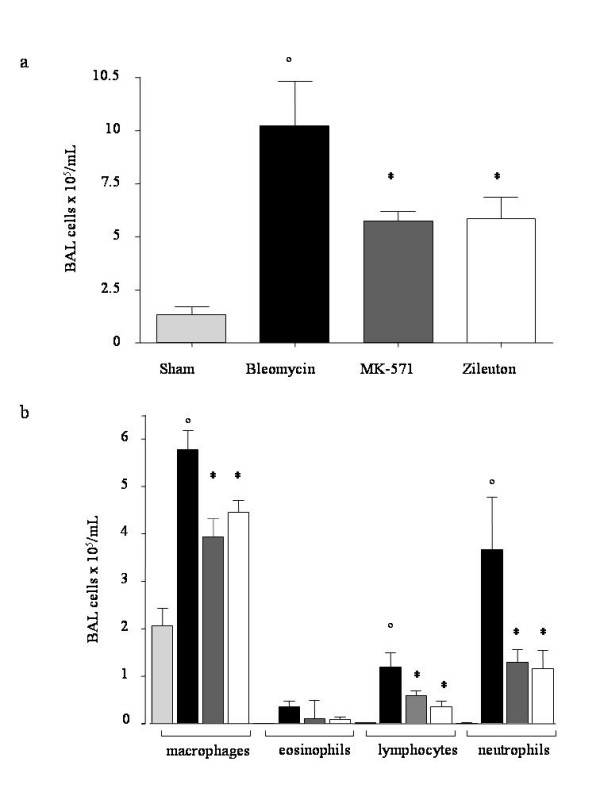
**Effect of pharmacological inhibition of leukotrienes on bleomycin-induced total (A) and differential cellularity (B) ofbronchoalveolar lavage (BAL)**. Total and differential cells counts for macrophages, lymphocytes, neutrophils and eosinophils per mL of BAL fluid are shown. Data, expressed as means ± SEM, are representative of 15 mice for each group. ° p < 0.001 vs. sham, *p < 0.05 vs. bleomycin.

BAL total cellularity significantly increased in bleomycin exposed animals (Fig. 5a). MK-571 and Zileuton groups showed a reduction in BAL cellularity when compared to bleomycin group.

Differential cell counts showed a similar profile across all of the sham groups. In the bleomycin group there was a significant increase of macrophages, lymphocytes and neutrophils compared to sham group.

MK-571 and Zileuton treated mice showed a decreased content of BAL inflammatory cells as evaluated on cytospins preparations (Fig. 5b). In these mice macrophages, lymphocytes and neutrophils were significantly reduced compared to bleomycin group.

## Discussion

Common pathologic features in interstitial lung diseases include the fibrosis of the interstitium, involve collagen, elastic and smooth muscle elements, architectural remodelling and chronic inflammation[18].

Lipid mediators are thought to be involved in lung fibrosis. Cysteinyl leukotrienes as well as LT-B4 are elevated in lung homogenates and bronchoalveolar lavage of patients with IPF [19-21]. In lung fibroblasts, leukotrienes stimulate collagen synthesis, chemotaxis, and transformation into myofibroblasts [22-24].

Observations on the role of leukotrienes *in vivo *come from the experimental model of bleomycin-induced lung fibrosis. Intratracheal instillation of the antitumour agent bleomycin is the most commonly used animal model for pulmonary fibrosis[25]. Earlier reports point out that the pathogenesis of bleomycin-induced fibrosis, at least in part, is mediated through the generation of reactive oxygen species which cause the peroxidation of membrane lipids and DNA damage[26].

Early attempts to target the arachidonic acid metabolism in this experimental model were performed using a pharmacological approach. Lpoxygenase inhibitor nordihydroguaiaretic acid was proved to attenuate bleomycin-induced lung fibrosis and to reduce both the macrophage infiltrate and the fibroblast growth factor release after bleomycin administration[27]. However, this compound is characterized by a non-specific action on arachidonic acid metabolism and has proved to possess a direct anti-oxidant activity [28]. Similarly, gamma-linolenic acid was able to suppress LT-B_4 _synthesis and lung damage in this model[29], but again its action is not limited to the arachidonic acid pathway[30].

Recently, leukotrienes pathway in this model has been dissected by genetically targeting the different enzymes responsible for the synthesis of eicosanoids. Indeed, lung fibrosis and inflammation were attenuated by the disruption of the gene encoding phospholipase A_2 _in this model[31]. Peters-Golden et al[32], demonstrated how 5-LO deficient mice were protected by bleomycin-induced lung fibrosis, thus confirming LT role in experimental pulmonary fibrosis. More recently, Beller and colleagues have demonstrated a role for LT-C_4 _synthase and for the cysteinyl leukotriene receptors in the pathogenesis of the fibrotic lung damage following bleomycin. Whereas the cys-LT_1 _receptor is involved in the acute damage, cys-LT2 receptor is thought to be responsible for the chronic injury following bleomycin administration[33,34].

However, it has to be underscored that murine alveolar macrophages present higher levels of cys-LTs than LTB4 with an inverted ratio between the two [35]. Thus, murine models are expected to exaggerate the importance of cys-LTs relative to what would occur in humans [36].

Considering that overproduction of 5-LO products occurs in the bleomycin animal model of lung fibrosis, and that previous studies on genetic knock out of different enzymes involved in leukotrienes synthesis have shown a significant protection from bleomycin induced fibrosis, we sought to assess the role of drugs that target the leukotriene pathway either at the synthetic step or at the receptor level.

In the current study, we used MK-571 as a specific cys-LT_1 _receptor antagonist[37]. This compound has similar biochemical and pharmacological properties to other antileukotrienes drugs such as montelukast, currently used to treat bronchial asthma and allergic rhinitis. Whereas Zileuton is a reversible 5-LO inhibitor approved for the treatment of asthma in humans. It is noteworthy that zileuton dose used in our experimental setup was very close to that clinically used in humans (1.5 times). On the other hand, it is not possible to estimate a relative dose for MK571, because of the unavailability of human studies with this particular compound.

Here we show a significant reduction of tissue damage in lungs of bleomycin-treated mice which received the treatment with both MK-571 or Zileuton. Not only did the matrix deposition evaluated histologically in lung sections of treated mice show a reduced degree of fibrosis, but also the alveolar architecture was preserved, indicating that the treatment with leukotrienes antagonists effectively prevented the bleomycin lung damage. In animals treated with MK-571 or Zileuton, lung edema and fall of body weight were virtually absent and inflammatory cells in BAL were significantly reduced. Moreover, we observed a significant reduction of leukocyte infiltration as assessed by the specific granulocyte enzyme MPO. Consistent with proinflammatory cell infiltrate and MPO activity we found that TNF-α was upregulated following intratracheal bleomycin administration. The TNF-α increase was completely abrogated in mice treated with MK-571 and Zileuton.

TNF-α is an "early-wave" cytokine, its role is recognized in a number of fibrotic human pulmonary pathologies[38]. It can induce apoptosis of respiratory epithelium which contributes to the alveolar damage in IPF. Moreover, there is evidence that TNF-α can upregulate the expression of the well known profibrotic cytokine TGF-β1 [39]. In fact, TNF-α blockade with either anti-TNF-α antibodies or TNF-α antagonists can inhibit fibrosis[40]. A cys-LT receptor 1 antagonist has been proved able to reduce the NF-kB activation and thus cytokines synthesis *in vitro*, and in particular TNF-α may be reduced secondarily to this effect[41]. The mechanisms of TNF-α pro-inflammatory activity are likely to involve both direct effects of TNF-α itself on regulation of adhesion molecule expression and induction of other cytokines and growth factors capable of mediating leukocyte chemotaxis and survival. Thus, it is conceivable that leukotrienes blockade results in a reduced inflammatory infiltrate in the lung following bleomycin administration and in an indirect effect on the active TGF-β levels in this model.

Similarly to TNF-α, we show that interleukin-1 (IL-1) is upregulated following bleomycin administration.

Interleukin-1β is one of the major extracellular proinflammatory cytokines, it is involved fibrotic process and is known to act synergistically with TNF-α [42].

Inhibition of IL-1β prevented the fibrotic reaction induced by bleomycin in mice[43], while its transient expression induces lung injury and pulmonary fibrosis in the late stages of the experimental setting [44].

We show that the IL-1β increase was almost abrogated in mice treated with MK-571 and Zileuton. This class of pharmacological agents has already shown the ability to suppress IL-1 secretion in cultured synovial tissue explants [45], potentially affecting the inflammatory cells infiltrate in tissues and thus the fibrotic response determined by the cascade of cytokines secreted following increased IL-1β release.

Finally, the beneficial effects given by the leukotrienes pharmacological blockade resulted in the abrogation of the mortality at 7 days after bleomycin.

To determine whether LTs play a causal role in fibrotic lung disease, we choose an interventional strategy to target both cysteinyl-LTs as well as LTB_4 _in the case of Zileuton or only cysteinyl-leukotrienes in the case of MK-571. This approach was selected on the basis of evidence that both classes of LTs are elevated in the bleomycin model as well as in human IPF [46]. Both classes of LTs have important actions that are fully relevant to inflammation as well as fibrogenesis.

Our data shows that both treatments granted a very similar degree of protection from bleomycin, with no evident differences between the two drugs in any of the parameters investigated. This might suggest on a first basis that leukotriene B_4 _have not a predominant role in mediating inflammation and fibrosis at least in bleomycin treated mice.

It has previously been demonstrated in a mouse model that cys-LT_2 _receptor is responsible for the fibrotic response to bleomycin administration by using a genetic approach to target this leukotrienes receptor [47]. We found that MK-571, a pharmacological cys-LT_1 _receptor antagonist, is able to block such response as well. Experimental gene disruption technique might generate a discrete number of variables that makes not feasible a straight parallel with a pharmacological study. On the other hand the receptor specificity of a pharmacological compound such as MK-571 is influenced by several factors related with pharmacological properties of the compound itself. In fact, although MK-571 is a specific cys-LT_1 _receptor antagonist, it possesses additional effects on leukotrienes methabolism. Indeed, this compound has been shown to inhibit the ubiquitously expressed multidrug resistance protein 1 (MRP1) as well [48]. MRP1 belongs to the ATP binding cassette transporter superfamily [49], its major physiological role is thought to be ATP-dependent transporter of LT-C_4_.

MRP1 knock out mice show a reduced inflammatory response induced by arachidonic acid due to impaired LT-C_4 _secretion [50]. Similarly, the specific MRP1 inhibitor MK571 is able to suppress LT-C_4 _transport *in vitro* [51].

MRP1 role in immunological responses is not limited to eicosanoids secretion. In example, MRP1 is implicated in T helper responses. MRP1 is constitutively expressed on Th2 cells while antigen or cytokine stimulation upregulates its expression on Th1 cells. MK571 has proved to depress T helper responses by decreasing the release of several cytokines such as IL-4, IFN-γ and TNF-α [52].

Considering this, it is tempting to speculate that the protective and anti-inflammatory effect of MK571 we observed could be linked to cys-LT1 receptor blockade as well as impaired cys-LT transport through MRP1. Further research is needed to address the relative role of the dual mechanism of action of MK571 in the bleomycin model of injury. The interest in MK571 action on LT-C_4 _transport is relatively recent. Nevertheless, a vast number of studies currently employ this compound as a cys-LT_1 _receptor antagonist both *in vitro *and *in vivo*.

In summary, we have provided the first evidence that antileukotrienes, drugs commonly used for their anti-inflammatory properties to treat asthma and allergic rhinitis, are able in the bleomycin animal model of lung fibrosis to attenuate the acute lung injury and the evolution of fibrotic lung lesions associated with the administration of this anticancer agent.

The beneficial activity of this pharmacological intervention was reflected on some favourable clinical outcomes such as reduced body weight loss, tissue edema and most notably mortality rate. Taken together, our data might further support the rationale for a clinical trial in interstial lung diseases as well as in other fibrotic diseases of the lung interstium including those associated with the usage of known causative drugs using antileukotriene compounds currently available for human use.
